# Supplementary material for: Perceptions on Extending the Use of Technology after the COVID-19 Pandemic Resolves: A Qualitative Study with Older Adults
Source: Int J Environ Res Public Health. 2022 Oct 29;19(21):14152. doi: 10.3390/ijerph192114152 (PMC9655749; doi:10.3390/ijerph192114152)
Supplement: Supplementary file 1 [file ijerph-19-14152-s001.zip › ijerph-1941560-supplementary.pdf]

Table S1. Portuguese cases interviews overview.

Case	How Was the Interview Conducted?	Total of Interview Sessions	Total Interview Time (min)
Case 1	Video – remote online	2	118.62
Case 2	Video – remote online	2	102.5
Case 3	Video – remote online	2	154.52
Case 4	Video – remote online	2	205.69
Case 5	Video – remote online	2	114.74
Case 6	Video – remote online	2	95.49
Case 7	Video – remote online	3	156.82
Case 8	Video – remote online	2	101.55
Case 9	Presential at home	2	140.22
Case 10	Video – remote online	2	142.48
**Total**		**21**	**1322.63**
